# Heat Transfer Enhancement in Parabolic through Solar Receiver: A Three-Dimensional Numerical Investigation

**DOI:** 10.3390/nano12030419

**Published:** 2022-01-27

**Authors:** Tayeb Fahim, Samir Laouedj, Aissa Abderrahmane, Sorour Alotaibi, Obai Younis, Hafiz Muhammad Ali

**Affiliations:** 1 Materials and reactive systems laboratory (LMSR), Djillali Liabes University, Sidi Bel Abbes 22000, Algeria; mrfahimtayeb@yahoo.com (T.F.); samirladz@yahoo.fr (S.L.); 2 Laboratoire de Physique Quantique de la Matière et Modélisation Mathématique (LPQ3M), Université Mustapha Stambouli de Mascara, Mascara 29000, Algeria; a.aissa@univ-mascara.dz; 3 Mechanical Engineering Department, College of Engineering and Petroleum, Kuwait University, P.O. Box 5969, Safat 13060, Kuwait; 4 Department of Mechanical Engineering, College of Engineering at Wadi Addwaser, Prince Sattam Bin Abdulaziz University, Wadi Addwaser 11991, Saudi Arabia; oubeytaha@hotmail.com; 5 Department of Mechanical Engineering, Faculty of Engineering, University of Khartoum, Khartoum 11111, Sudan; 6 Mechanical Engineering Department, King Fahd University of Petroleum and Minerals, Dhahran 31261, Saudi Arabia; hafiz.ali@kfupm.edu.sa; 7 Interdisciplinary Research Center for Renewable Energy and Power Systems (IRC-REPS), King Fahd University of Petroleum and Minerals, Dhahran 31261, Saudi Arabia

**Keywords:** Monte Carlo Ray Trace, nanofluids, heat transfer, obstacles, tube receiver, parabolic trough collector

## Abstract

Parabolic trough collectors (PTC) are one of the most established solar concentrating systems which have been used in a wide variety of applications. Enhancing their performance is critical to establish them as a viable technology. Internal obstacles are an intriguing way for improving the collector’s performance. However, the usage of obstacles results in increasing pressure loss. The purpose of this research is to numerically explore the impact of introducing obstacles to the receiver tube of a parabolic trough collector on heat transmission in PTCs and its overall thermal performance. The first part analyzed the effects of geometrical parameters, orientation angle (α = 45°, 90° or 135°), and spacing of obstacles (P/D = 1, 2, or 3) on the fluid motion, heat transfer, and performance. Then, a non-uniform heat flow was applied to the absorber’s outer surface. The effects of nanoparticles type, temperature profile, and heat transfer performance of three different nanofluids (Cu/thermal oil, Al_2_O_3_/thermal oil, andTiO_2_/thermal oil) were studied in the second part. The simulation results show that, the friction factor increased when P/D decreases, and that the absorber tube with obstacles discs (α = 90°) and P/D = 2 achieved the best thermal performance. Additionally, increasing the concentration of solid nanoparticles in thermal oil improves heat transmission, and the Cu nanofluid has the greatest Nusselt number.

## 1. Introduction

In the current global environment, the use of renewable sources is of prime importance due to growing negative effects of environmental pollution and climate change [1,2,3,4,5]. Solar energy is probably the only green source of energy that can meet the increasing global energy demands, and it has been grabbing the most attention with numerous research papers being published every year [6,7,8,9]. Like other power generation methods, solar energy has various drawbacks and challenges that must be solved to improve efficiency and minimize costs [10,11]. Due to their superior performance and effectiveness in this type of application, solar power plants use parabolic trough collector (PTC) for large-scale energy generation [12,13,14]. A parabolic trough collector’s reflector concentrates incident solar radiation on the lower half peripheral of the absorber tube. The glass envelope exposes the receiving tube’s perimeter to direct sunlight on the other side. The receiver tube absorbs the concentrated solar rays as heat, and then transfers to the employed liquid circulating through it. The thermal efficiency of a PTC is frequently evaluated by numerically simulating the complex optical-thermal conversion using two-dimensional or three-dimensional models [15], with the solar heat radiation flux and absorber wall temperature assumed to be uniform. Much important research has been done, and MCRT(Monte Carlo ray tracing), the ray-tracing method, and both finite volume and element methods (FVM and FEM) have been created in recent years to depict this process. For computing the impact of solar irradiance on the absorber tube, several optical models have been created by employing ray tracing or MCRT, such as the works of Mao et al. [16], Petrasch [17], He and Cheng et al. [18,19], and Grena [20]. Some models combine MCRT and FVM, such as those developed by, He and Cheng et al. [21,22], while others combine MCRT and FEM, such as those developed by Eck et al. [23] and Wang et al. [24]. However, referring to experimental data is still required in this field because of the lack of a faultless model [25,26,27].

The main notion of the researched concepts for increasing thermal performance is to increase the heat transport rate between the absorber and the fluid. This lowers the temperature of the absorber and reduces thermal losses, resulting in increased thermal efficiency. One option that has received much attention in the literature is nanoliquid as working liquid in PTC [28]. Metal-based nanoparticles such as Al_2_O_3_, Fe, Fe_2_O_3_, TiO_2_, Cu, and SiO_2_ are dispersed in a working fluid (typically thermal oil or water) to form these fluids [29]. The resultant mixture (nanofluid) has an enhanced density, heat capacity, and thermal conductivity, which improves the system’s thermal performance. Numerous research [30,31] have looked into using water-based nanofluids in PTCs and have found improvements of up to 4%.

Other researchers employed devices called turbulator and various inserts to enhance PTC performance [32,33,34,35]. Jaramillo et al. [36] investigated the conditions under which twisted tape inserts can help with performance. Researchers showed that when these implants were used at low motion rates with a twist ratio close to one, the thermal performance rate inside the PTC was improved. Amina et al. [37] compared the integration of two internal ribs in the lower section of a PTC absorber tube. For various Reynolds numbers, they discovered that rectangular and triangular ribs result in a thermal improvement index of 1.15 to 1.45. Kumar and Reddy [38] established a 3D numerical model for thermal analysis of PTC using a porous disc receiver. Thermal fluid characteristics, solar radiation, and receiver design all affected the total heat collection. Therminoil-VP1 was used as the working fluid, and the renormalization group (RNG) K– turbulent model was used. They found a 64.3% rise in Nusselt; however, there were considerable pressure losses. Ghasemi et al. [39] explored the use of many permeable discs in the absorber of a PTC. They discovered that a larger porous disc and a more significant number of porous discs lead to greater thermal enhancement. Gong et al. [40] investigated numerically the heat transfer enhancement of PTC with pin-fin arrays inserting. They concluded that the overall heat transfer performance of PTC with inserts is higher than that of conventional PTC. Huang et al. [41] examined internally dimpled absorbers; their results demonstrate that the performance evaluation criterion is close to 1.3. Seyed and Akbar [42] studied numerically the effect of porous rings on performance of PTC; they declared that thermal performance of heat transfer fluid in the porous ring absorber tube was improved compared to that of the smooth absorber tube. Moreover, the Nusselt number enhanced with the increase of the size of ring. Amina et al. [43] compared the single phase with the VOF (Volume of Fluid Method) and mixture two-phase models for forced convection flow of alumina/Dowtherm—a nanofluid in a tube side of PTC fitted with two longitudinal rectangular fins; their results demonstrate that the mixture two-phase model gives better results compared to the homogenous model and the use of compound technique leads to enhanced heat transfer. Bellos et al. [44] presented a numerical study of thermal enhancement in a converging-diverging PTC receiver tube; they found that the wavy inner geometry creates more turbulent conditions in the flow and it decreases the pressure losses in the collector. Amina et al. [45] established numerically the heat transfer improvement inside a PTC tube fitted with central corrugated insert; their results show that the Nusselt number of finned absorber varied from 1.3 to 1.8 times in comparison to that of smooth tube, and the fin shape has a remarkable effect on heat transfer characteristics. Bellos et al. [46] studied numerically the impact of internal longitudinal fins on PTC; they remarked that fins with higher length leads to higher efficiency and higher-pressure losses. Aggrey et al. [47] presented a numerical investigation of thermal performance of receiver for a parabolic trough collector (PTC) with perforated plate inserts. Their results show that the use of inserts improves the thermodynamic performance of the receiver by minimizing the entropy generation rates, and described the dependence of the Nusselt number and friction factor on the spacing and size of the insert. Wang et al. [48] investigated numerically the heat transfer enhancement in the receiver tube of a direct steam generation system with parabolic trough by inserting metal foams; they reported the significant effect of the layout and dimensionless height of metal foams on the thermal performance, whereas the porosity of the foam proved to have a slight influence on the heat transfer. Cheng et al. [49] carried out a numerical study of heat transfer enhancement by unilateral longitudinal vortex generators inside the PTC receiver. They illustrated that the average Nusselt number and average friction factor increase with increasing each geometric parameter, whereas the thermal loss decreases with the increase of each geometric parameter. Saha et al. [50] investigated pressure drop and heat transfer characteristics in a circular tube equipped by regularly spaced twisted tape elements; they declared that pinching of place rather is a better property from thermo-hydraulic performance.

Following a study of prior research, it is determined that earlier work may be enhanced by combining heat transfer methods with obstacles and nanofluids. The combination of a passive and active techniques can give the system a great improvement in heat transfer. This investigation is conducted with the aim to increase the amount of thermal energy absorbed by the working fluid in PTC system. This study uses impediments to perform three-dimensional numerical simulations on the receiver tube of a PTC with a circumferentially non-uniform heat flux with distribution obtained by applying MCRT (Monte Carlo ray tracing) method. The first half examines the impacts of geometrical parameters, orientation angle, and obstacle spacing on fluid motion and heat transfer. Furthermore, the receiver tube’s thermal performance was investigated to determine the best values for the introduced obstructions. Finally, the second portion looked into the impact of nanoparticle type on the temperature profile and heat transfer performance.

## 2. Physical Model

**Figure 1:**
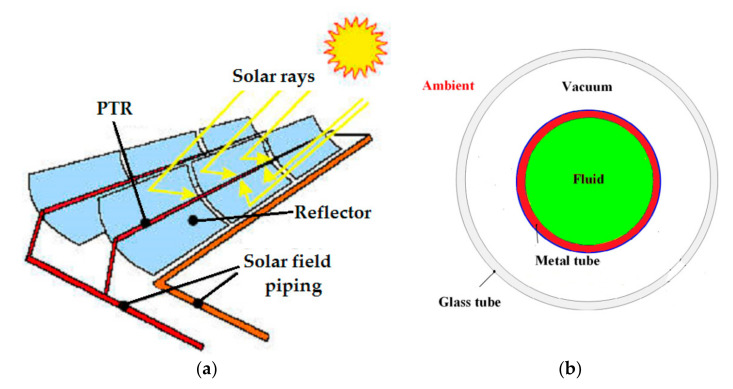
(**a**) Parabolic trough collector (PTC). (**b**) Schematic diagram of cross section of the parabolic trough receiver (PTR) [51]. Reproduced with permission from [51]. Elsevier, 2015.

Figure 1 shows the PCT blueprint with information on the tube receiver. Most of the incoming solar rays were guided to the PTR’s bottom edge by the solar collector; however, the upper half of the PTR (parabolic trough receiver) was exposed to non-concentrated solar rays. A metal tube, usually made of stainless steel, is surrounded by a glass cover. The gap between the glass cover and the metal tube was kept vacuumed in order to reduce heat loss. Table 1 shows the geometric characteristics of the PTR.

**Figure 2:**
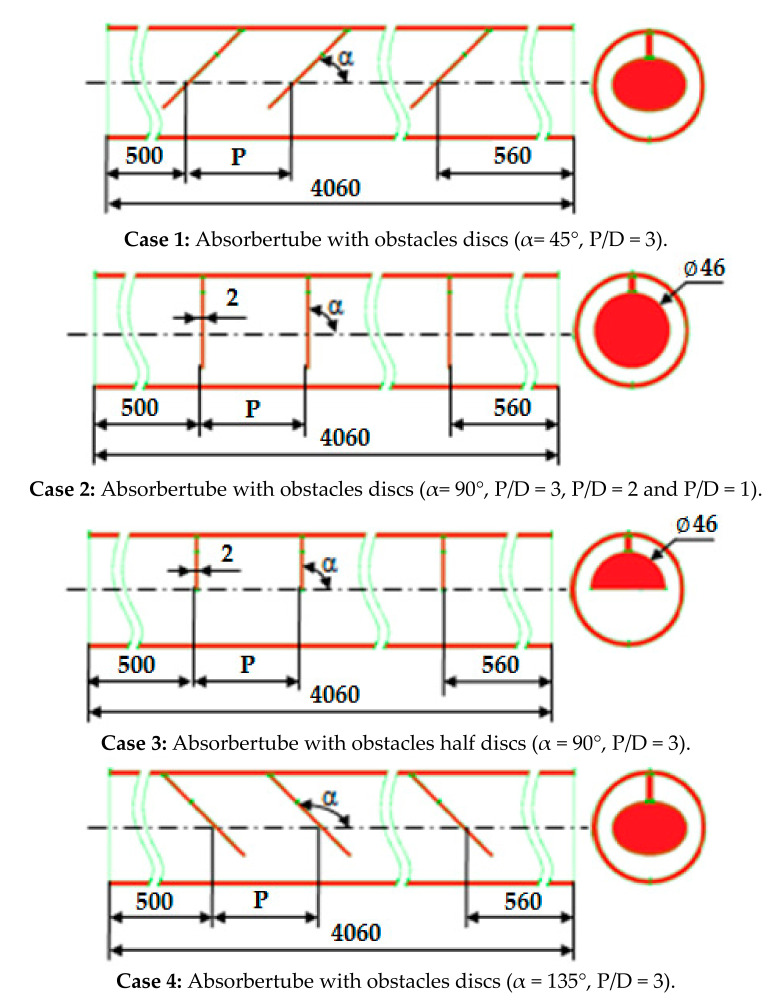
Physical model of absorber tube with obstacles.

The effect of placing barriers in the metal tube of PTC on heat transmission was investigated numerically in this work. The distance separating the tube inlet and outlet is (L = 4.06 m), the tube’s inner diameter is (D), the obstacle’s diameter is (d), the distance between the obstacles (P), and the obstacles inclination angle (α). Figure 2 shows the models configurations used in this work; for the four cases we used different orientation angles and obstacle spacing to show their effects on heat transfer enhancement.

### 2.1. Boundary Conditions

- Fluid inlet;

v_x_ = v_in_, v_y_ = v_z_ = 0 m/s,

T_f_ = T_in_ = 400 K (L = 0, 0° ≤ φ ≤ 360°).

**Figure 3:**
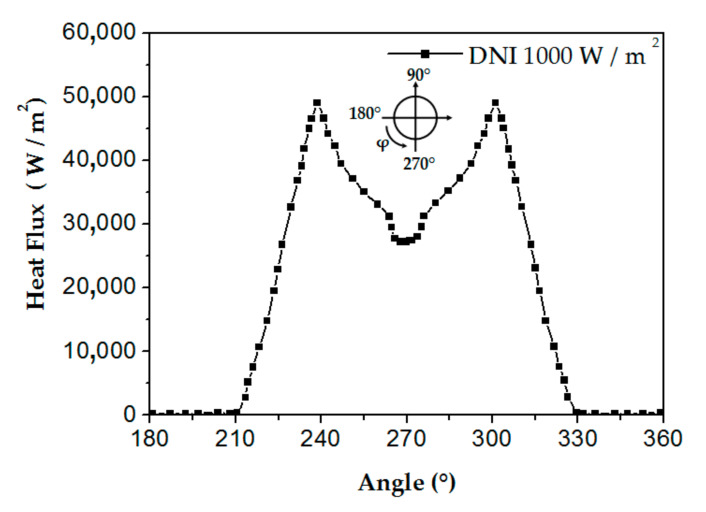
Heat flux distribution fluctuation throughout the absorber tube’s bottom half perimeter [54]. Reproduced with permission from [54]. Elsevier, 2013.

- Wall boundary condition;

The uniform heat flux q_t_ is applied to the metal tube’s upper half periphery:

q_t_ = DNI × TGE × AMT = 1000 × 0.95 × 0.96 = 912 W/m^2^ (0 ≤ L ≤ 4.06 m, 0° ≤ φ ≤ 180°).

where DNI is the solar irradiance, TGE is the glass envelope transmissivity, and AMT is the metal tube absorptivity. Hachicha et al. [54] calculated the concentrated solar irradiation qcal (Figure 3). The q**_b_** heat flux is applied to the tube receiver’s bottom half periphery:

q**_b_** = q_cal_(0 ≤ L ≤ 4.06 m, 180° ≤ φ ≤ 360°).

**Figure 4:**
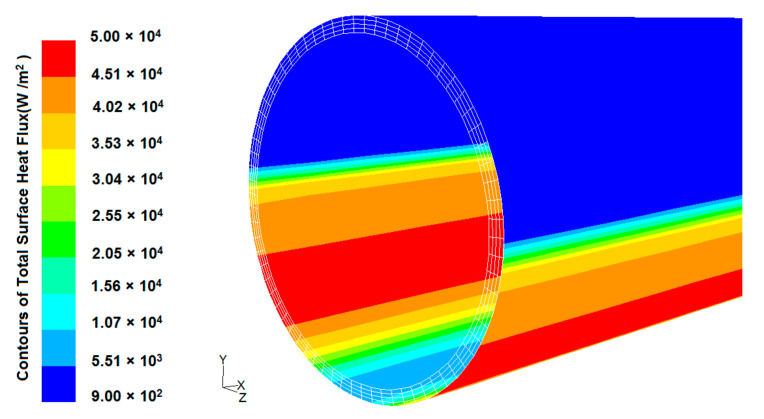
Heat flux distribution on an absorber tube’s surface (the present study).

- Fluid outlet: fully developed conditions.
- In this study, the outer absorber’s wall receives a non-uniform heat flux obtained by using MCRT technique, and taking the DNI of 1000 W/m^2^, the local concentration ratio distribution results are illustrated in Figure 3 and the heat flux distribution on an absorber tube’s surface is shown in Figure 4.

### 2.2. Thermo-Physical Characteristics of the HTF

In the PTR, thermal oil D12 is widely utilized as a heat transfer fluid. Thermo-physical properties are temperature (°C) dependent [55]:

ρ = −0.696982 × T − 0.131384 × 10^−3^ × T^2^ − 0. 209079 × 10^−5^ × T^3^ + 776.257 (1)

C_p_ = 2.01422 × 10^3^ + 0.00386884 × T + 2.05029 × 10^−3^ × T^2^ − 1.12621 × 10^−5^ × T^3^ + 3.86282 × 10^−8^ × T^4^ (2)

λ = 0.112994 − 0.00014781 × T − 1.61429 × 10^−7^ × T^2^ (3)

ν = exp((530.944/T + 146.4) − 2.68168)(4)

### 2.3. Numerical Method

**Figure 5:**
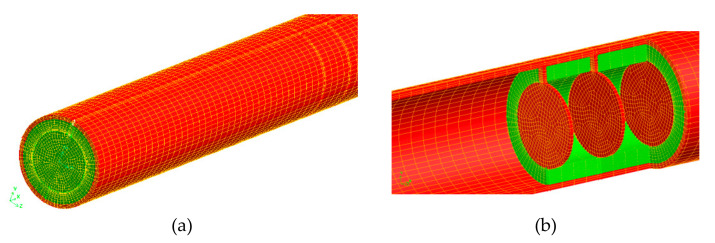
Three-dimensional Hexa grid use in this study. (**a**) global mesh of the absorber. (**b**) detailed mesh near obstacles.

The governing equations were computed using the VOF method using the segregated implicit solver of the CFD software (R 17.1) using the first-order formulation. The software ANSYS-FLUENT (Release 17.1) was then utilized to create and mesh the geometrical three-dimensional model, see Figure 5. The hexagonal mesh was applied for volume mesh. Forced convection in the absorber tube was simulated using the RNG k–model with typical wall functions. The model is validated based on published reports of experimental data [56]. Different mass flow rates are calculated in the range of Reynolds number 18860-81728.

## 3. Results and Discussion

For validate purposes, the numerical results of temperature values for smooth tube were compared with those given by Roldán et al. [56].

Roldán et al. [56] employed thermocouples to investigate the thermal performance of PTC with a length of 4.06 m. Using the view of the thermocouples implanted in the PTR, the temperature of the metal tube’s exterior surface was measured. Table 2 summarizes the detailed experimental data for each of the six scenarios that were investigated. In addition, the highest and minimum temperatures on the metal tube’s fluid outflow surface were also recorded for model validation.

Standard k–ε model, realizable k–ε model, and RNG k–ε model were utilized for model validation to determine which model would suit further numerical data. Table 3 shows the temperature variations between experimental and computational results in detail, with the relative error (*δ*) defined as:

(5) $$\delta =\frac{\left|T_{\exp}-T_{\mathrm{num}}\right|}{T_{\exp}}\times 100\%$$

**Figure 6:**
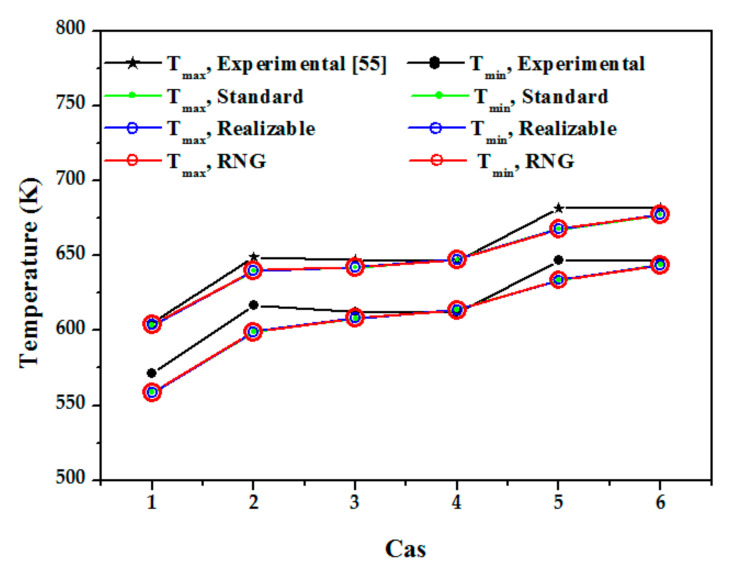
Temperature on outlet surface of tube receiver [56]. Reproduced with permission from [56]. Elsevier, 2013.

Figure 6 shows the temperature on outlet surface of tube receiver in different cases for different turbulence models. The numerical results demonstrate that each model closely fits the thermal performance test by Roldán et al. [56]. Furthermore, the average deviations are the smallest when the RNG k-model is used. On the fluid outflow surface of the tube receiver, Figure 6 shows the temperature contrast between experimental measurements [56] and numerical simulations using the RNG k-model. As a result, the RNG k-model is utilized in the numerical simulations that follow.

In order to justify the accuracy as well as the stability of the numerical results, extensive calculations have been made to determine the total number of grid points that generate an appropriate array result that will be appropriate to determine flux and thermal field. Table 4 presents the evolution of average Nusselt number as a function of cell number for a variation of Reynolds number between 10^4^ and 10^6^. 

### 3.1. Obstacle Form and Orientation on Heat Transfer Effect

The results were carried out in this case for a receiver setup with obstacles. The heat transfer capabilities of the PTR heat transfer fluid are investigated by passing it through a metal tube. It is defined as follows: the Nu_avg_, Re and heat transfer coefficient (h):

(6) $$\mathrm{Nu}=\frac{h.D}{\lambda }$$

(7) Re=D.vν

(8) $$h=\frac{q\prime \prime }{T_{t,a}-T_{f,a}}$$

Furthermore, for turbulent flow, the Darcy friction factor [57] is defined as follows

(9) $$f=\frac{2.\Delta P.D}{L.\rho .v^{2}}$$

After balancing the pressure and shear forces, the same expression takes the form:

(10) $$f=\frac{8.\tau_{w}}{\rho .v^{2}}$$

Gnielinski [58] proposed the following equation for calculating the Nusselt number smooth tube:

(11) $${\mathrm{Nu}}_{D}=\frac{(f/8)\left(R_{\mathrm{eD}}-1000\right)\mathrm{Pr}}{1+12.7{\left(f/8\right)}^{1/2}({\mathrm{Pr}}^{2/3}-1)}$$

For 3000≤Re≤5×106 and 0.5≤Pr≤2000

The Petukhov correlation for friction factor is given by [59]:

(12) $$f=\;{\left(0.790{\;\mathrm{lnRe}}_{D}-1.64\right)}^{-2}$$

For 3000≤Re≤5×106

**Figure 7:**
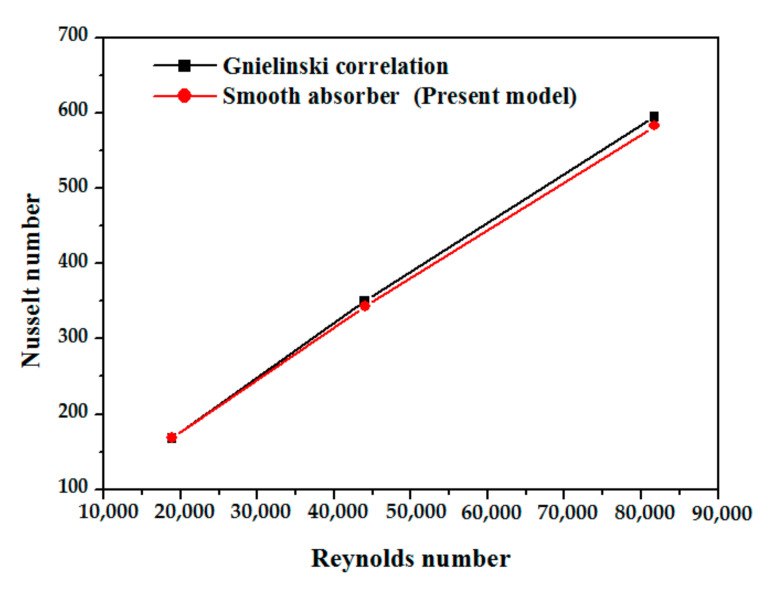
Nussle number with Gnielinski correlation [58].

**Figure 8:**
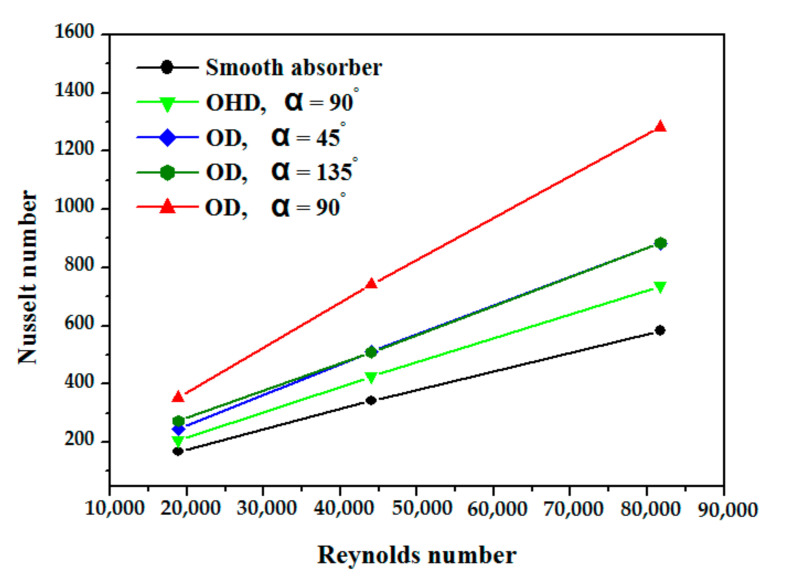
Nu variation in the absorber vs. Reynolds number (P/D = 3).

In terms of the Nusselt number, Figure 7 shows a comparison of the smooth absorber’s Nusselt number with the Gnielinski correlation. The model reasonably agrees with the Gnielinski equation for all Reynolds numbers, which is a good result where the maximum deviation is less than 7.8% and the minimum deviation is approximately 0.18%. As shown in Figure 8, for the five examples under consideration, the fluctuation of Nusselt number is caused by changes in Reynolds number (Re), which has the values of 18860 (v = 0.15 m/s), 44007 (v = 0.35 m/s), and 81725 (v = 0.65 m/s) while utilizing thermal oil D12 as working fluid. The Nusselt number increases nearly linearly in relation to the Reynolds number, this augmentation is due to increasing heat transfer area by inserting obstacles. The vortex flow was produced due to fluid mixing provided by the obstacles, and higher turbulent intensity at such a high Re causes the destruction of the thermal boundary layer. The greatest increase is observed for absorber tubes with obstacles discs at an angle of 90 degrees. Compared to the reference case with the smooth absorber, the mean Nusselt number increases by 129%. The use of obstacles discs (at angles of 45° and 135°) is the second most effective example, with a mean Nusselt number improvement of 95%, while the use of obstacles half discs at angles of 90° and 135° results in the lowest Nusselt number improvement, which is close to 79%. 

**Figure 9:**
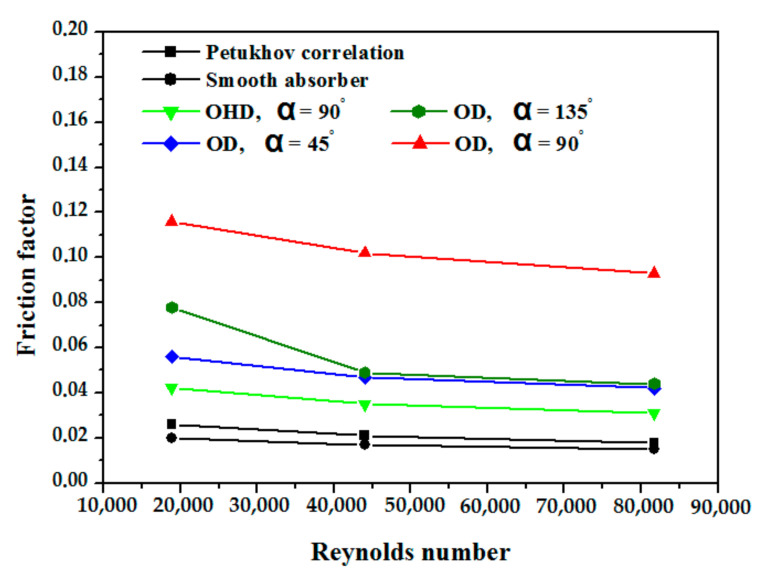
Variation in absorber tube’s friction factor vs. Reynolds number (P/D = 3).

Figure 9 demonstrates that the friction factor of the smooth case is the lowest of all the examples considered in this study. The use of obstacles discs (with an angle of 90°) results in the highest friction factor, followed by obstacles discs (with an angle of 45° and 135°) and obstacles half discs (with an angle of 90°) in the second and third cases, respectively. These higher values are the results of the swirling flow induced by the inserts that act like an obstacle.

**Figure 10:**
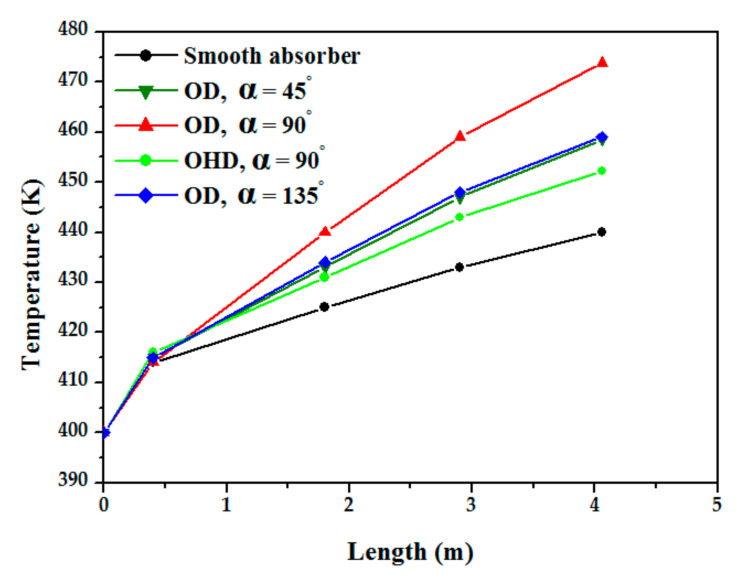
Variation in absorber tube’s average temperature and without obstacles (Re = 44007, *v* = 0.35 m/s).

Comparing the friction factor derived in this study in the smooth absorber with the Petukhov correlation shows that it corresponds to reality. The same figure demonstrates that the highest and smallest discrepancies between our numerical results and Petukhov’s correlation are 15% and 11%, respectively. On sectional planes (y-axis and z-axis) over the length of the absorber tube with and without obstructions, Figure 10 depicts the average temperature distribution for heat transfer fluid. The temperature reaches its maximum at the exit, and higher temperatures are obtained in the absorber tube with obstruction discs (α = 90°) case than in the other cases.

### 3.2. Effect of Obstacles Spacing on Heat Transfer

**Figure 11:**
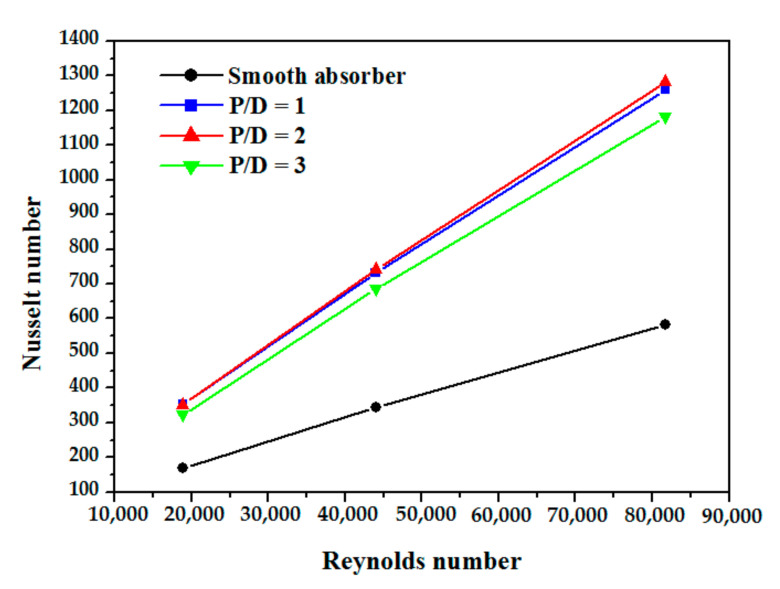
Nusselt number variation vs. Re number at different obstacle spacing.

The effect of distance between two consecutives obstacles was carried out by varying the longitudinal distance (P/D). As illustrated in Figure 11, the results show that Nusselt number increases by decreasing the distance between obstacles which influences the thermal transport and the detachment and reattachment of the boundary layer. 

**Figure 12:**
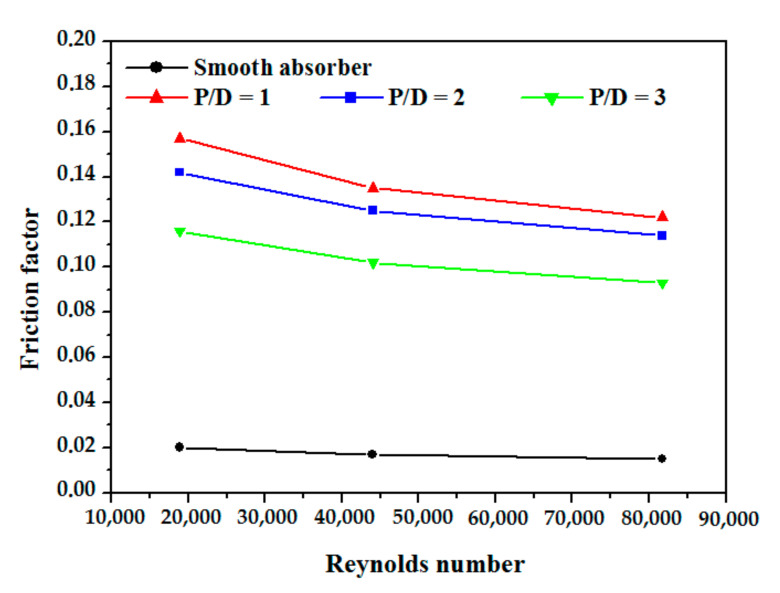
Friction factor variation vs. Re number at different obstacle spacing.

Figure 12 depicts the fluctuation of the friction factor for the absorber tube with and without obstacles as the Reynolds number increases for various P/D values for the absorber tube with and without obstacles. The friction factor increases with decreasing P/D due to the increase in the number of barriers with declining P/D.

### 3.3. Thermal Performance Analysis

In order to improve heat transfer efficiency, it is required to assess both heat transfer and flow resistance at the same time. As a result, the performance evaluation criterion (PEC), which is defined as follows [57], is a universal assessment criterion that captures the total performance of a heat transfer unit. The thermal performance criteria was calculated which is defined as the ration of the dimensionless Nusselt number and the dimensionless friction factor given by:

(13) $$\mathrm{PEC}\;=\frac{\mathrm{Nu}/{\mathrm{Nu}}_{0}}{{\left(f/f_{0}\right)}^{1/3}}$$

**Figure 13:**
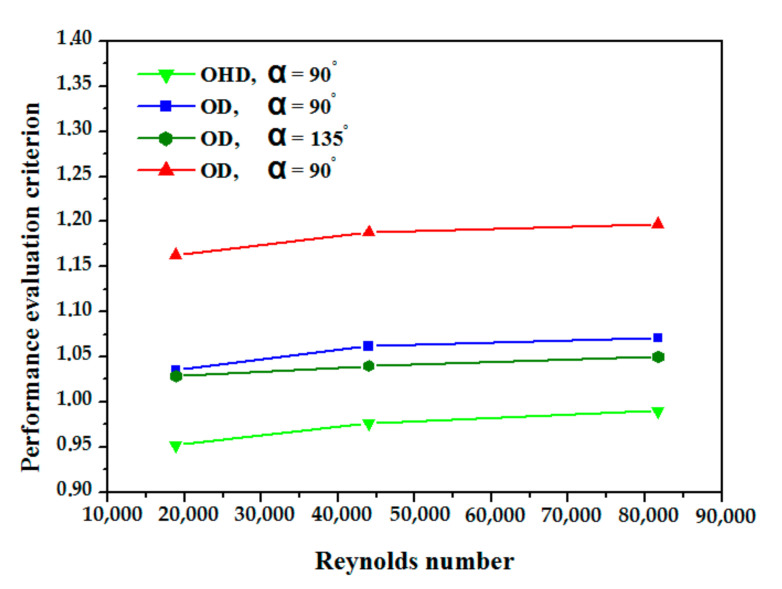
Performance evaluation criterion variation vs. Re number of the examined cases.

The smooth absorber case is represented by the letters (Nu_0_) and (f_0_). The variation of performance evaluation criteria (PEC) is shown in Figure 13, when the PEC values are higher than 1 it means that the inserts have a good effect on heat transfer, in this study PEC values vary from 0.95 to 1.20, it is significant that the obstacles offer a heat transfer enhancement than of the smooth tube.

**Figure 14:**
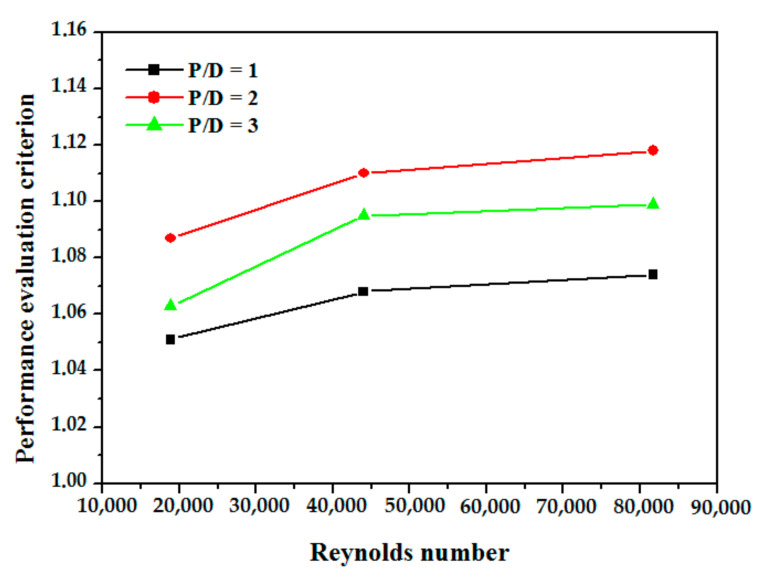
Performance evaluation criterion variation vs. Re number at different values of p/d.

The performance evaluation of PEC with Re for different values of P/D is represented in Figure 14 which demonstrates the effect of obstacle’s spacing on heat transfer performance. Results have shown that PEC have the maximum value for P/D equal to 2, it is attributed to the influence of this spacing on thermal transport and the reattachment of the boundary layer.

## 4. Effect of Different Types of Nanoparticles

This section aims to study the effects of nanoliquids on heat transfer during a flow through an absorber tube with disc obstacles (α = 90° and P/D = 2) of a solar collector cylinder -parabolic. Three different types of nanoparticles that are Cu, Al_2_O_3,_ and TiO_2_, dispersed in a base fluid (thermal oil D12), these particles are used at a concentration of (ϕ=0.01) and a nanoparticle diameter of 10 nm. A two-phase mixture model is employed to study the forced convective heat transfer of nanoliquid. The physical properties of the nanofluid; the mass density ρ_nf_ [60], the viscosity μ_nf_ [61], thermal conductivity k_nf_ [62], and the specific heat Cp_nf_ [63], are given by the following equations:

(14) $$\rho_{\mathrm{nf}}=\rho_{P}\phi +\left(1-\phi \right)\rho_{f}$$

(15) $$\mu_{\mathrm{nf}}=\frac{\mu_{f}}{{\left(1-\phi \right)}^{2.5}}$$

(16) $$\lambda_{\mathrm{nf}}=\lambda_{f}\frac{\lambda_{P}+2\lambda_{f}-2\phi \left(\lambda_{f}-\lambda_{P}\right)}{\lambda_{P}+2\lambda_{f}+\phi \left(\lambda_{f}-\lambda_{P}\right)}$$

(17) $${\mathrm{Cp}}_{\mathrm{nf}}=\frac{\rho_{p}{\mathrm{Cp}}_{p}\phi +\rho_{f}{\mathrm{Cp}}_{f}2\phi \left(1-\phi \right)}{\rho_{\mathrm{nf}}}$$

**Figure 15:**
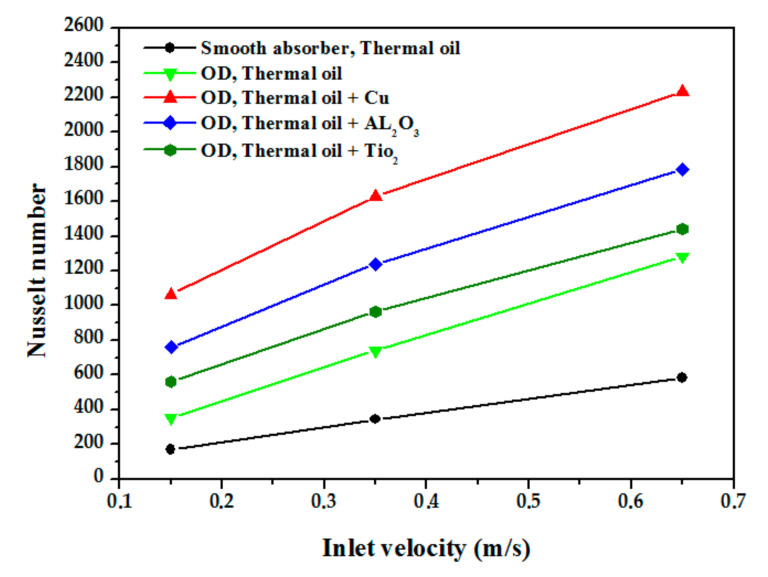
Nusselt number of cases examined for various inlet velocities.

**Figure 16:**
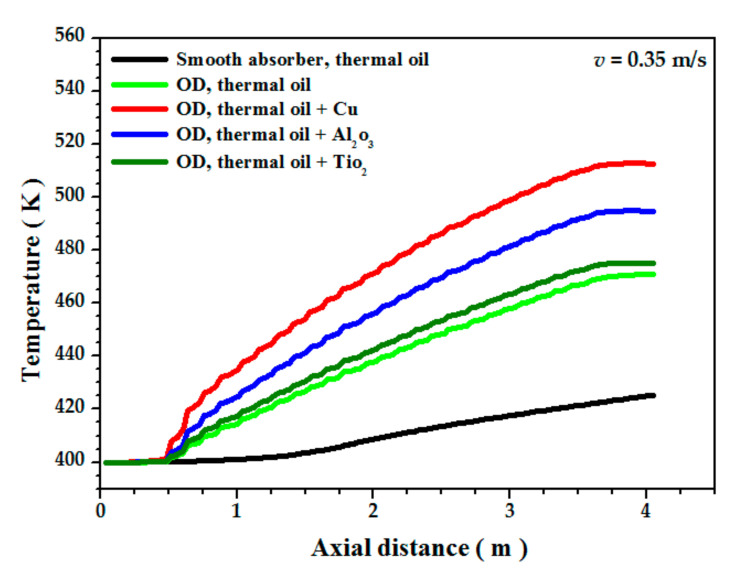
Axial evolution of nanofluid temperature for inlet velocity (*v* = 0.35 m/s).

**Figure 17:**
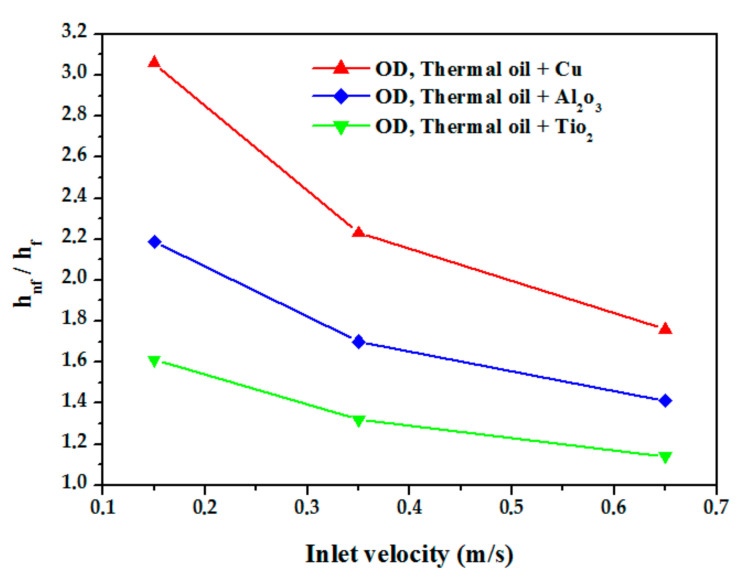
Evolution of the relative heat transfer coefficient.

Figure 15 represents the variation of the Nu as a function of inlet velocity for different nanofluids. It is noted that the Nu of the nanoliquids is greater than that of the base liquid (thermal oil D12), and it increases with increasing the inlet velocity which increases turbulence intensity and has a direct influence on heat transfer enhancement. Lower values of the Nu are obtained in the case of the TiO_2_ nanofluid/thermal oil, average values for Al_2_O_3_/thermal oil, and the largest values for Cu/thermal oil which is due to the thermal performance of Cu/thermal oil compared toother nanofluids; using Cu nanoparticles increases the thermal performance of the fluid by five times. The axial variation of the average temperature along a vertical plane passing through the main axis is illustrated in Figure 16. The average temperature increases linearly from the inlet to the outlet of the absorber tube. The figure shows that the highest temperature obtained in the nanofluid is by the Cu/thermal oil, followed by Al_2_O_3_/thermal oil, then TiO_2_/thermal oil. Figure 17 shows the relative heat transfer coefficients evolution, defined as the ratio of the convective heat transfer coefficient of the nanofluids to that of the base fluid (thermal oil D12) as a function of the inlet velocity. The relative heat transfer coefficient decreases with the inlet velocity of the fluid. This figure also shows the effect of the type of nanoparticles on the variation of the heat transfer coefficient. It was observed that the thermal performance rate is maximum for the nanofluid Cu/thermal oil.

## 5. Conclusions

In this paper, the influence of using different obstacles on the thermal performance of parabolic through solar receiver is numerically explored. In order to use the most suitable numerical model for this simulations, the results of three different numerical models were compared to previous results. The following remarks could be concluded from work.

- The RNG k- ε model is adopted to compute the turbulent motion in the absorber tube of PTR as it gives the minimum error when compared to the literature.
- The obtained friction factor and Nusselt number showed good agreement with Petukhov and Gnielinski equation correlations.
- The usage of obstacles in the absorber tube has a favorable influence on Nusselt number, and adverse effect on friction factor.
- Compared to the reference case, the highest improvement in Nusselt number was 129% which was achieved by the obstacles discs (α = 90°), followed by 95% for the obstacles discs (OD, α = 45° and α = 135°), while the obstacles half discs (α = 90°) achieved the lowest improvement of only 79%.
- The friction factor is inversely proportional to the value of P/D.
- The optimum thermal performance is obtained when the absorber tube with obstacles discs (α = 90°) and P/D = 2.
- Heat transfer is significantly augmented when using nanofluids.
- The use of Cu nanoparticles resulted in the best thermal performance compared to Al_2_O_3_ and TiO_2_.

## Figures and Tables

**Table 1:** Optical parameters of the PTR [52,53].

Parameter	Values
Reflector/Absorber Length	4.06 m
Metal Tube Inner Diameter	0.067 m
Metal Tube Outer Diameter	0.07 m
Glass Cover Inner Diameter	0.117 m
Glass Cover Outer Diameter	0.12 m
Parabolic Trough Concentrator Reflectivity	0.9
Glass envelope Transmissivity	0.96
Metal Tube Absorptivity	8027 kg/m^3^
Metal Tube Thermal Conductivity	20 W/m.k
Metal Tube Specific heat	500 J/kg.k

**Table 2:** Thermal performance test conducted by Roldán et al. [56].

Case	P_in_ (MPa)	T_in_ (K)	DNI (W/m^2^)	ṁ (Kg/s)
1	6.0	557.5	838	0.73
2	6.1	598.1	761	0.62
3	6.0	607.3	635	0.55
4	6.0	613	627	0.56
5	6.0	632.9	635	0.55
6	6.0	643	627	0.56

**Table 3:** Temperature variations between experimental and computational results in detail, with the relative error.

Case	1	2	3	4	5	6
Experimental results “Roldàn et al. [56]”						
T_max_	604.7	649.1	647	646.8	681.6	681.5
T_min_	571.4	616.6	612.2	612.2	646.8	646.4
Standard k-ɛ model						
T_max_	603.5	639.92	642.21	647.46	667.75	677.39
δ	0.18	1.41	0.74	0.1	2.03	0.6
δ_moyen_	0.84					
T_min_	558.61	599.1	608.13	613.82	633.74	643.83
δ	2.23	2.83	0.66	0.26	2.01	0.39
δ_moyen_	1.39					
Realizable k-ɛ model						
T_max_	603.6	639.95	642.23	647.48	667.78	677.42
δ	0.18	1.4	0.73	0.1	2.02	0.59
δ_moyen_	0.83					
T_min_	558.59	599.09	608.12	613.81	633.73	643.82
δ	2.24	2.83	0.66	0.26	2.02	0.39
δ_moyen_	1.4					
RNG k-ɛ model						
T_max_	603.69	640.02	642.29	647.54	667.84	677.48
δ	0.16	1.39	0.72	0.11	2.01	0.58
δ_moyen_	0.82	599.06	608.1	613.79	633.7	643.82
T_min_	558.59	599.06	608.1	613.79	633.7	643.82
δ	2.24	2.84	0.66	0.25	2.02	0.4
δ_moyen_	1.4					

**Table 4:** Mesh effect on the average Nusselt number.

N_cells_					
	294,600	307,200	330,400	354,400	
Re	Nu				|δ_max_|
10^4^	148.123	150.021	149.613	150.461	1.57%
10^5^	222.104	226.371	226.719	229.004	3.10%
10^6^	259.121	258.223	259.942	260.781	0.64%

## Data Availability

All data are available upon request from any of the authors.

## Nomenclature

C_p_	Specific heat capacity, (J/kg.K)	ν	Kinematic viscosity, (m^2^/s)
D	Absorber tube inner diameter, (m)	ρ	Density, (kg/m^3^)
d	Diameter of obstacle, (m)	τ_w_	Wall shear stress, (Pa)
f	Friction factor	ϕ	Nanoparticle volume fraction, %
h	Heat transfer Coefficient, (W/m^2^.K)	φ	Circumferential angle, (°)
L	Tube length, (m)	**Subscripts**	
ṁ	Mass flow reat, (kg/sec)	avg	Average
Nu	Nusselt number	b	Bottom
P	Distance between the obstacles, (m)	cal	Calculated
P	Pressure, (Pa)	D	Hydraulic diameter
∆P	Pressure drop, (Pa)	exp	Experiment
Pr	Prandtl number	f	Fluid
q	Heat flux, (W/m^2^)	in	Inlet
Re	Reynolds number	max	Maximum
T	Temperature, (K)	min	Minimum
v	Velocity, (m/s)	n_f_	Nanofluid
**Greek symbols**		n_p_	Nanoparticle
α	Inclination angle of the obstacles, (°)	t	Top
δ	Deviation, (%)	x, y, z	Cartesian coordinates
ε	Rate of dissipation of turbulent kinetic energy	**Abbreviation**	
*k*	Turbulent kinetic energy, (J/kg)	DISS	Direct Solar Steam
λ	Conductivity, (W/m.K)	DNI	Direct normal irradiance
HTF	Heat transfer fluid	PEC	Performance evaluation criteria
LCR	Local concentration ratio	PTC	Parabolic trough collector
MCRT	Monte Carol ray-tracing	PTR	Parabolic trough receiver
OD	Obstacles discs	RNG	Renormalization-group
OHD	Obstacles half discs		
